# Integrated analysis of isorhamnetin-associated targets in chronic pancreatitis and pancreatic cancer: immune-infiltration associations and antitumor effects in pancreatic cancer cells

**DOI:** 10.3389/fimmu.2026.1902080

**Published:** 2026-09-04

**Authors:** Ming Xu, Renren Sun, Wei Li, Lei Wei, Shuai Li, Xiaoxiao Li, Wei Hou, Huiping Xie, Zelin Xu

**Affiliations:** 1 The School of Basic Medical Sciences, Qilu Medical University, Zibo, China; 2 The School of Basic Medical Sciences, North China University of Science and Technology, Tangshan, China

**Keywords:** chronic pancreatitis, EGFR, immune infiltration, isorhamnetin, network pharmacology, pancreatic cancer

## Abstract

**Objective:**

Chronic pancreatitis (CP) creates an inflammatory pancreatic microenvironment and is an established risk factor for pancreatic cancer (PC). However, immune-inflammatory links between CP and PC remain unclear. Isorhamnetin (ISO), a natural flavonoid with anti-inflammatory and antitumor activities, may influence pancreatic inflammatory signaling. This study investigated ISO-associated molecular networks shared by CP and PC, examined the relationships between prioritized targets and transcriptome-estimated immune-cell infiltration, and evaluated the effects of ISO in pancreatic cancer cells.

**Methods:**

CP- and PC-associated targets were collected from disease databases, and ISO targets were obtained from drug-target prediction platforms. Disease- and ISO-related targets were analyzed using protein–protein interaction networks, functional enrichment, and hub-gene screening. The expression, prognostic relevance, diagnostic performance, and immune infiltration associations of hub genes were evaluated using TCGA-PAAD, GTEx, Kaplan–Meier Plotter, ROC analysis, and CIBERSORTx. Molecular docking and 100 ns molecular dynamics simulations assessed ISO–target binding stability. Two-sample Mendelian randomization (MR) explored potential causal associations between genetically proxied circulating levels of the six hub proteins and CP or PC risk. The effects of ISO on proliferation, wound closure, apoptosis, and target protein expression were examined in PANC-1 and BXPC-3 cells.

**Results:**

372 overlapping CP- and PC-associated targets and 48 ISO-related targets were identified. EGFR, AKT1, MMP9, BCL2, TNF, and IL6 were screened as hub genes. Functional enrichment indicated targets were involved in inflammatory responses, cytokine-related signaling, apoptosis regulation, epithelial proliferation, pancreatic cancer-related pathways, and PI3K-Akt signaling. Hub-gene expression was correlated with CIBERSORTx-estimated immune-cell fractions, including activated natural killer cells and several T-cell subsets. Molecular docking and molecular dynamics simulations provided preliminary support for ISO–target interactions and complex stability. Across the 12 prespecified protein–outcome combinations, MMP9–CP was the only nominal IVW association. *In vitro*, ISO reduced the viability and wound closure of PANC-1 and BXPC-3 cells, promoted apoptosis, and decreased EGFR, AKT1, BCL2, and TNF-α protein expression in a concentration-dependent manner.

**Conclusion:**

ISO may influence CP- and PC-associated molecular networks through multitarget regulation involving EGFR, AKT1, MMP9, BCL2, TNF, and IL6. These findings provide hypothesis-generating computational, genetic, and cellular evidence supporting investigation of ISO in pancreatic inflammation-associated tumor biology. However, immune regulatory effects, MMP9/IL6 protein modulation, and EGFR/AKT pathway activation require further experimental validation.

## 1 Introduction

CP is a progressive fibroinflammatory disease of the pancreas caused by multiple genetic and environmental factors. It is characterized by irreversible pancreatic parenchymal injury, fibrosis, and functional impairment, ultimately leading to intractable abdominal pain and pancreatic exocrine and endocrine insufficiency, thereby severely compromising patients’ quality of life (1). Epidemiological studies have shown that the incidence and prevalence of CP are increasing worldwide, while effective curative treatments remain unavailable (1, 2). More importantly, CP is one of the most important risk factors for PC and markedly increases the likelihood of PC development (3, 4). PC is a highly aggressive malignancy of the digestive system, with a 5–year survival rate persistently below 10%. Most patients are diagnosed at an advanced stage and have already lost the opportunity for curative surgery, resulting in a substantial disease burden (5–7). Currently, clinical management of CP and PC mainly focuses on symptom relief, complication control, surgery, chemotherapy, radiotherapy, and other conventional approaches. However, early diagnosis remains difficult, therapeutic efficacy is limited, and chemoresistance continues to represent a major clinical challenge. Therefore, exploring the molecular mechanisms underlying the transition from CP to PC and identifying novel intervention targets and therapeutic strategies are of critical scientific significance and urgent clinical relevance.

In recent years, rapid advances in network pharmacology and bioinformatics have provided powerful tools for systematically elucidating complex disease–drug interactions (8, 9). By constructing “drug–target–disease” networks, this approach enables the prediction of potential drug targets and molecular mechanisms at a holistic and systems level, overcoming the limitations of the traditional “single-target, single-drug” research paradigm. It has become an important strategy for the modernization of traditional Chinese medicine and mechanistic studies of natural products (10). Complementary to network pharmacology, molecular docking and molecular dynamics simulation can evaluate the binding modes and stability of drug molecules with target proteins at the atomic level, thereby providing preliminary computational validation for virtual screening results (11, 12). In addition, MR analysis, which uses genetic variants as instrumental variables to infer causal relationships between exposures and outcomes, can effectively reduce confounding bias and reverse causality in conventional observational studies, providing a higher level of causal evidence for the identified key targets (13).

ISO is a natural flavonoid compound widely found in plants such as sea buckthorn and Ginkgo biloba, and it has attracted considerable attention due to its diverse biological activities. Previous studies have shown that ISO possesses significant anti-inflammatory, antioxidant, antitumor, and cardioprotective effects (14). In the context of antitumor activity, ISO has been shown to inhibit the proliferation of various cancer cells, induce apoptosis, and suppress migration and invasion by regulating multiple key signaling pathways, including PI3K/AKT, MAPK, and NF-κB (15–17). Meanwhile, the pathophysiological processes of both CP and PC involve chronic inflammation, oxidative stress, and uncontrolled cell proliferation, all of which are biological processes targeted by ISO. Therefore, ISO represents a highly promising candidate molecule for intervention in CP-related pathological progression and PC development. However, whether ISO directly affects CP and PC, as well as its potential molecular targets and precise mechanisms, remains to be systematically investigated.

Compared with previous studies that mainly investigated the direct anti-proliferative or pro-apoptotic effects of ISO in pancreatic cancer cells, the novelty of the present study lies in its integrated CP-PC-oriented analytical framework. Specifically, we combined network pharmacology to identify ISO-associated CP-PC targets, molecular docking and molecular dynamics simulation to evaluate ISO–target binding stability, Mendelian randomization to explore potential causal relationships between hub genes and disease risk, immune infiltration analysis to assess tumor microenvironment-associated signatures, and cellular experiments to validate selected phenotypic and protein-level changes. This strategy provides a broader systems-level perspective on how ISO may be linked to inflammation-associated pancreatic tumor biology rather than focusing solely on tumor-cell autonomous effects.

## 2 Materials and methods

### 2.1 Identification of CP- and PC-related disease targets

To comprehensively identify disease-related target genes associated with CP and PC, the GeneCards, DisGeNET, OMIM, and Therapeutic Target Database (TTD) databases were searched using “chronic pancreatitis” and “pancreatic cancer” as keywords. These databases were selected because they provide complementary disease-target information. GeneCards provides relevance-score-based gene–disease associations, DisGeNET integrates curated and literature-derived disease–gene evidence, OMIM contains manually curated disease-associated genes, and TTD provides therapeutic target annotations. To balance sensitivity and specificity, a GeneCards relevance score > 1 was used to retain genes with at least moderate disease relevance. For DisGeNET, disease-specific thresholds were applied according to the number and distribution of available disease-associated genes. The gene–disease association score was set at ≥ 0.02 for CP-related genes and ≥ 0.01 for PC-related genes, in order to retain potentially relevant CP-associated genes while reducing the inclusion of low-confidence disease associations. Relevant gene information from OMIM and TTD was subsequently integrated, and duplicate entries were removed to improve data accuracy. Finally, 499 CP-related genes and 875 PC-related genes were obtained.

### 2.2 Identification of ISO-related drug targets

Potential targets of ISO were predicted using the Comparative Toxicogenomics Database (CTD), PharmMapper, ETCM, Similarity Ensemble Approach (SEA), and SwissTargetPrediction databases. A total of 58, 75, 53, 82, and 103 potential drug target genes were obtained from these databases, respectively. All results were integrated and deduplicated for subsequent analyses.

### 2.3 GO and KEGG enrichment analyses

To investigate the potential biological functions of overlapping CP- and PC-associated genes, Gene Ontology (GO) functional enrichment analysis and Kyoto Encyclopedia of Genes and Genomes (KEGG) pathway enrichment analysis were performed on the 372 overlapping genes. Gene symbols were first converted into Entrez Gene IDs for subsequent analysis. GO and KEGG enrichment analyses were conducted using the clusterProfiler package, version 4.10.0, in R software, version 4.3.2, with *P* < 0.05 considered statistically significant. The ggplot2 package was used for visualization. GO results were presented as bar plots, including biological process (BP), cellular component (CC), and molecular function (MF). KEGG enrichment results were displayed as bubble plots. In addition, to further explore the mechanisms by which ISO participates in CP-PC-related pathological processes, GO and KEGG enrichment analyses were performed on the 48 intersecting targets shared by ISO and CP-PC. GO results were visualized as bar plots, whereas KEGG results were presented as Sankey bubble plots.

### 2.4 Construction of the protein–protein interaction network

The 372 overlapping CP- and PC-associated genes were imported into the STRING database to construct a protein–protein interaction (PPI) network. The species was set as *Homo sapiens*, and the minimum required interaction confidence score was set at 0.40. The TSV-formatted network file generated by STRING was then downloaded and imported into Cytoscape software, version 3.9.1 (18), for visualization and topological analysis. The same approach was used to construct the PPI network for the 48 ISO-CP-PC intersecting targets.

### 2.5 Identification of hub genes

Topological analysis of the PPI network was performed using the CytoHubba plugin in Cytoscape. Four algorithms, namely Degree, Edge Percolated Component (EPC), Maximal Clique Centrality (MCC), and Betweenness, were used to score the nodes. The top 10 candidate hub genes ranked by each algorithm were selected, and the intersection of genes identified by the four algorithms was used to determine the final hub genes. For the 48 ISO-CP-PC intersecting target genes, the same four algorithms were used for hub gene screening, and the Molecular Complex Detection (MCODE) plugin was used to identify key functional modules. Finally, key hub genes were determined by integrating the results of the four algorithms and MCODE analysis.

### 2.6 Bioinformatics analyses and associations with estimated immune-cell infiltration

Based on The Cancer Genome Atlas pancreatic adenocarcinoma cohort (TCGA-PAAD) and Genotype-Tissue Expression (GTEx) databases (19, 20), the expression, prognostic value, and immune infiltration characteristics of the six hub genes in PC were analyzed. First, the mRNA expression levels of the hub genes were compared between normal pancreatic tissues and PC tissues. Kaplan–Meier Plotter was then used to perform survival analysis and evaluate the association between hub gene expression and overall survival (OS). Receiver operating characteristic (ROC) curve analysis of the hub genes was performed using the pROC package in R, and visualization was conducted using ggplot2. Furthermore, the CIBERSORTx immune infiltration algorithm was used to analyze the correlations between hub gene expression and immune cell infiltration, and the results were presented as a heatmap.

### 2.7 Molecular docking analysis of ISO binding to target genes

The two-dimensional structure of ISO was obtained from the PubChem database, with Compound CID 5281654, and converted into PDB format using Open Babel software, version 2.3.2 (21). Receptor protein structures were obtained from the Protein Data Bank (PDB), including AKT1 (PDB ID: 7NH5), MMP9 (1GKC), TNF (2AZ5), EGFR (3POZ), BCL2 (1GJH), and IL6 (1ALU). PyMOL software, version 2.3.4 (22), was used to remove water molecules and original ligands from the protein structures. AutoDockTools, version 1.5.7 (23), was used to add hydrogen atoms and charges to the receptor proteins. The ligand and receptor proteins were then converted into PDBQT format, and molecular docking analysis was performed using AutoDock Vina, version 1.1.2 (24). Finally, Discovery Studio 2019 and PyMOL were used to visualize the molecular docking results in two-dimensional and three-dimensional formats.

### 2.8 Molecular dynamics simulation analysis of predicted binding models

YASARA software, version 10.3.16 (25), was used to perform molecular dynamics simulation (MDS) of ISO–protein complexes. The small-molecule ligands and protein receptors were first subjected to energy minimization, followed by 100 ns all-atom MDS. The simulation time step was set at 1 fs, and a total of 1 × 10^7^ steps were performed. After the simulation, trajectory data were extracted using the built-in tools of YASARA and analyzed using GraphPad Prism 8. Root mean square deviation (RMSD) and root mean square fluctuation (RMSF) were calculated to evaluate the conformational stability of the complexes and changes in residue flexibility.

### 2.9 MR analysis

Two-sample MR analysis was performed for all 12 prespecified protein–outcome combinations, comprising six hub-protein traits (BCL2, AKT1, EGFR, IL6, MMP9, and TNF) evaluated against two outcomes (CP and PC). Single nucleotide polymorphisms (SNPs) were used as instrumental variables (IVs). Exposure and outcome summary statistics were obtained from the IEU OpenGWAS database, and MR analysis was conducted using the TwoSampleMR package in R. The exposure datasets were protein-level GWAS summary statistics: BCL2 (prot-a-236), AKT1 (prot-a-70), EGFR (prot-a-909), IL6 (prot-a-1539), MMP9 (prot-a-1921), and TNF (prot-a-3029). These proteomic GWAS datasets were derived from the INTERVAL study and included 3,301 participants of European ancestry (26). The CP outcome was chronic pancreatitis from FinnGen (finn-b-K11_CHRONPANC; 1,737 cases and 195,144 controls; total n = 196,881; European ancestry) (27), and the PC outcome was pancreatic cancer (ebi-a-GCST90018893; 1,196 cases and 475,049 controls; total n = 476,245; European ancestry) (28). Thus, the MR exposures represent genetically proxied circulating protein levels rather than mRNA expression. Detailed information on the databases, platforms, software versions, and access dates used for the computational analyses is provided in **Supplementary Table 1**, and detailed characteristics of the MR exposure and outcome datasets are provided in **Supplementary Table 2**. The inverse variance weighted (IVW) method was used as the primary estimator; MR-Egger, weighted median (WM), simple mode, weighted mode, maximum likelihood, and radial IVW methods were used as complementary or sensitivity analyses. Odds ratios (ORs) and 95% confidence intervals (CIs) were calculated, with OR > 1 indicating a risk effect and OR < 1 indicating a protective effect. Because this MR component was exploratory and hypothesis-generating, no formal multiple-testing correction was applied across the 12 primary IVW tests. Accordingly, nominal two-sided P values are reported for all 12 combinations, and *P* < 0.05 was used only to identify signals for further investigation rather than to establish definitive causal evidence. Complete primary IVW results for all 12 combinations are provided in **Supplementary Table 3**. For instrumental variable selection, SNPs associated with the corresponding protein exposure were extracted from the IEU OpenGWAS database using a significance threshold of *P* < 5 × 10^-6^. Linkage disequilibrium clumping was performed using r² < 0.001 within a 10,000-kb window to ensure the independence of instrumental variables. SNPs unavailable in the outcome dataset or removed during exposure–outcome harmonization were excluded. For the MMP9–CP analysis, 14 SNPs were retained after harmonization. Instrument strength was assessed using the F-statistic, calculated as β²/SE² based on the SNP–exposure association. SNPs with F-statistics > 10 were considered sufficiently strong instruments. Heterogeneity among instrumental variables was assessed using Cochran’s Q test based on the IVW and MR-Egger methods, with Q-test *P* < 0.05 indicating significant heterogeneity. Horizontal pleiotropy was evaluated using the MR-Egger intercept test, with *P* < 0.05 indicating potential directional horizontal pleiotropy. Leave-one-out sensitivity analysis was further performed by sequentially removing each SNP to determine whether the MR estimate was driven by a single instrumental variable. The results were presented using scatter plots, forest plots, funnel plots, and leave-one-out plots.

### 2.10 Cell culture

Human PC cell lines PANC-1 and BXPC-3 were purchased from Procell Life Science & Technology Co., Ltd. (Wuhan, China). PANC-1 cells were cultured in high-glucose Dulbecco’s Modified Eagle Medium (DMEM; HyClone), while BXPC-3 cells were cultured in RPMI-1640 medium (Gibco). Both culture media were supplemented with 10% fetal bovine serum (FBS; Gibco). Cells were maintained in a humidified incubator at 37 °C with 5% CO_2_. ISO was purchased from Chengdu Gelipu Biotechnology Co., Ltd. and dissolved in dimethyl sulfoxide (DMSO; OriGen) to prepare stock solutions according to the manufacturer’s instructions. For all cellular experiments, the final DMSO concentration was maintained at 0.1% (v/v) in all experimental groups, including the 0, 20, 40, and 80 μM ISO groups. The 0 μM ISO group contained 0.1% DMSO and served as the vehicle control.

### 2.11 Cell proliferation and apoptosis assays

Cell proliferation was assessed using the Cell Counting Kit-8 assay (CCK-8; Dojindo, China). PANC-1 and BXPC-3 cells were seeded into 96-well plates at a density of 1 × 10^4^ cells per well. After 24 h of culture, the medium was replaced with medium containing different concentrations of ISO, namely 0, 20, 40, and 80 μM, and cells were incubated for another 24 h. The culture medium was then discarded, and 100 μL of medium containing 10% CCK-8 reagent was added to each well. After incubation at 37 °C for 1.5 h, absorbance was measured at 450 nm using a microplate reader (Tecan).

Cell apoptosis was detected using an Annexin V-FITC/PI double-staining kit (Yeasen). After treatment with different concentrations of ISO for 24 h, cells were digested with trypsin without EDTA and collected. Cells were resuspended in 100 μL of binding buffer, followed by the addition of 5 μL Annexin V-FITC and 10 μL PI staining solution. After incubation for 15 min at room temperature in the dark, 400 μL of binding buffer was added before analysis using a flow cytometer (BD Canto II).

### 2.12 Wound healing assay

A wound-healing assay was performed to assess wound closure in PANC-1 and BXPC-3 cells after ISO treatment. Cells were seeded into 6-well plates and cultured until confluence reached over 90%. The cell monolayer was then washed with phosphate-buffered saline, and a straight wound was created vertically using a sterile 200 μL pipette tip. After removal of detached cells, the medium was replaced with medium containing different concentrations of ISO, namely 0, 20, 40, and 80 μM. The final DMSO concentration was matched at 0.1% (v/v) across all groups. Images of wound areas were captured using an inverted phase-contrast microscope at 0 h and 24 h. Wound areas were measured using ImageJ software, and the wound closure rate was calculated using the following formula: Wound closure rate (%) = [1 − (wound area at 24 h/wound area at 0 h)] × 100%. Because wound-healing assays may be influenced by both cell proliferation and migration, the results were interpreted as changes in wound closure rather than definitive evidence of proliferation-independent migration ability. All experiments were independently repeated three times.

### 2.13 Western blot analysis

PANC-1 and BXPC-3 cells were seeded into 6-well plates and treated with different concentrationsof ISO, namely 0, 20, 40, and 80 μM, for 24 h. Total proteins were extracted using RIPAlysis buffer (Beyotime), and protein concentrations were determined using a BCA protein assay kit(Beyotime). Equal amounts of protein, 30 μg per sample, were separated by 12.5% SDS-PAGE and transferred onto polyvinylidene fluoride membranes (PVDF; Beyotime). The membranes were blocked with 5% skimmed milk in TBST at room temperature for 1 h. The membranes were incubated overnight at 4 °C with the following primary antibodies purchased from Hangzhou Huaan Biotechnology Co., Ltd.: GAPDH (Cat. No. EM1101, dilution 1:1000), EGFR (Cat. No. ET16044-44, dilution 1:1000), AKT1 (Cat. No. ET1609-51, dilution 1:1000), BCL2 (Cat. No. ET1702-53, dilution 1:1000), and TNFα (Cat. No. ER65189, dilution 1:1000). After washing three times with TBST, the membranes were incubated with horseradish peroxidase-conjugated secondary antibodies (Beyotime) at room temperature for 1 h. Protein bands were visualized using enhanced chemiluminescence reagent (ECL; Beyotime), and images were acquired using a ChemiDoc imaging system (Bio-Rad).

### 2.14 Statistical analysis

**Figure 1:**
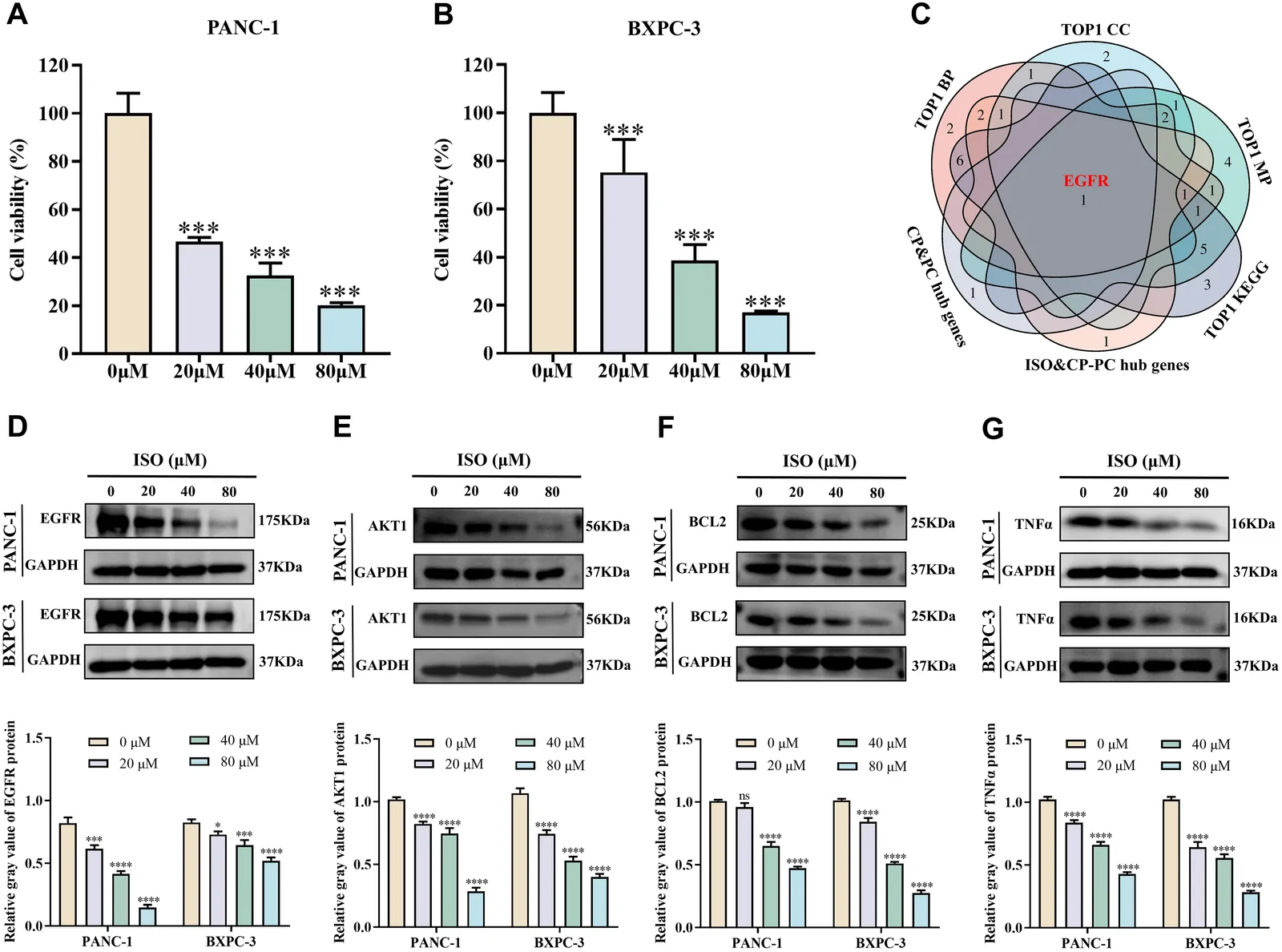
Effects of ISO on PC cell viability and related protein expression. **(A, B)** CCK-8 assays showing changes in the viability of PANC-1 and BXPC-3 cells after treatment with different concentrations of ISO (0, 20, 40, and 80 μM). **(C)** Intersection analysis of the TOP1 target, CP-PC hub genes, and ISO-CP-PC hub genes, identifying EGFR as the key target. **(D–G)** Western blot analysis of EGFR, AKT1, BCL2, and TNFα protein expression levels in PANC-1 and BXPC-3 cells after treatment with different concentrations of ISO, together with densitometric analysis. GAPDH was used as the internal control. Compared with the 0 μM group, *P < 0.05, ***P < 0.001, ****P < 0.0001; ns, not significant. Data are representative of three independent experiments, n = 3 biological replicates, mean ± SD.

**Figure 2:**
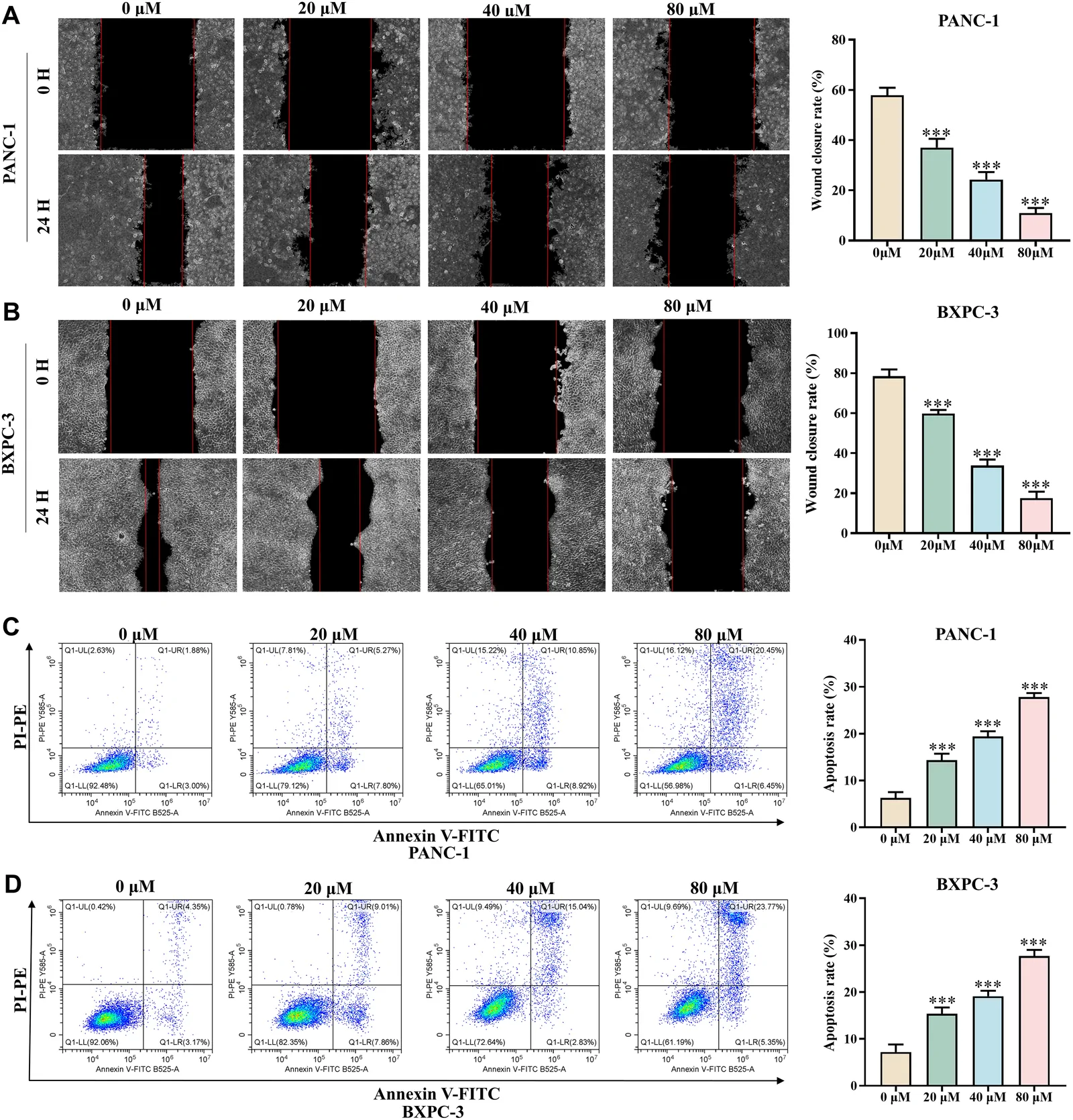
Effects of ISO on PC cell wound closure and apoptosis. **(A, B)** Wound-healing assays showing wound closure in PANC-1 and BXPC-3 cells after treatment with different concentrations of ISO, together with statistical analysis of wound closure rates. **(C, D)** Annexin V-FITC/PI double-staining flow cytometry detecting apoptosis in PANC-1 and BXPC-3 cells after treatment with different concentrations of ISO, together with statistical analysis of apoptosis rates. Compared with the 0 μM group, ***P < 0.001. Data are representative of three independent experiments, n = 3 biological replicates, mean ± SD.

All cellular experiments were performed as three independent biological replicates. For the CCK-8 assay, each treatment condition included three technical wells per independent experiment. Wound-healing and apoptosis assays were independently repeated three times, and Western blotting was performed using protein samples from three independent biological replicates; densitometric values were normalized to GAPDH. Statistical analyses were performed using GraphPad Prism 8.0.2, and data are presented as mean ± standard deviation (SD). Before parametric testing, normality and variance homogeneity were assessed. Two-sided Student’s t-test was used for two-group comparisons when parametric assumptions were satisfied. For the four-group *in vitro* dose comparisons (0, 20, 40, and 80 μM ISO) shown in **Figure 1** and **2**, one-way analysis of variance (ANOVA) was performed, followed by Dunnett’s multiple comparisons test to compare each ISO-treated group (20, 40, and 80 μM) with the 0 μM vehicle-control group. All tests were two-sided unless otherwise stated, and *P* < 0.05 was considered statistically significant.

## 3 Results

### 3.1 Identification and enrichment analysis of overlapping CP- and PC-associated targets

**Figure 3:**
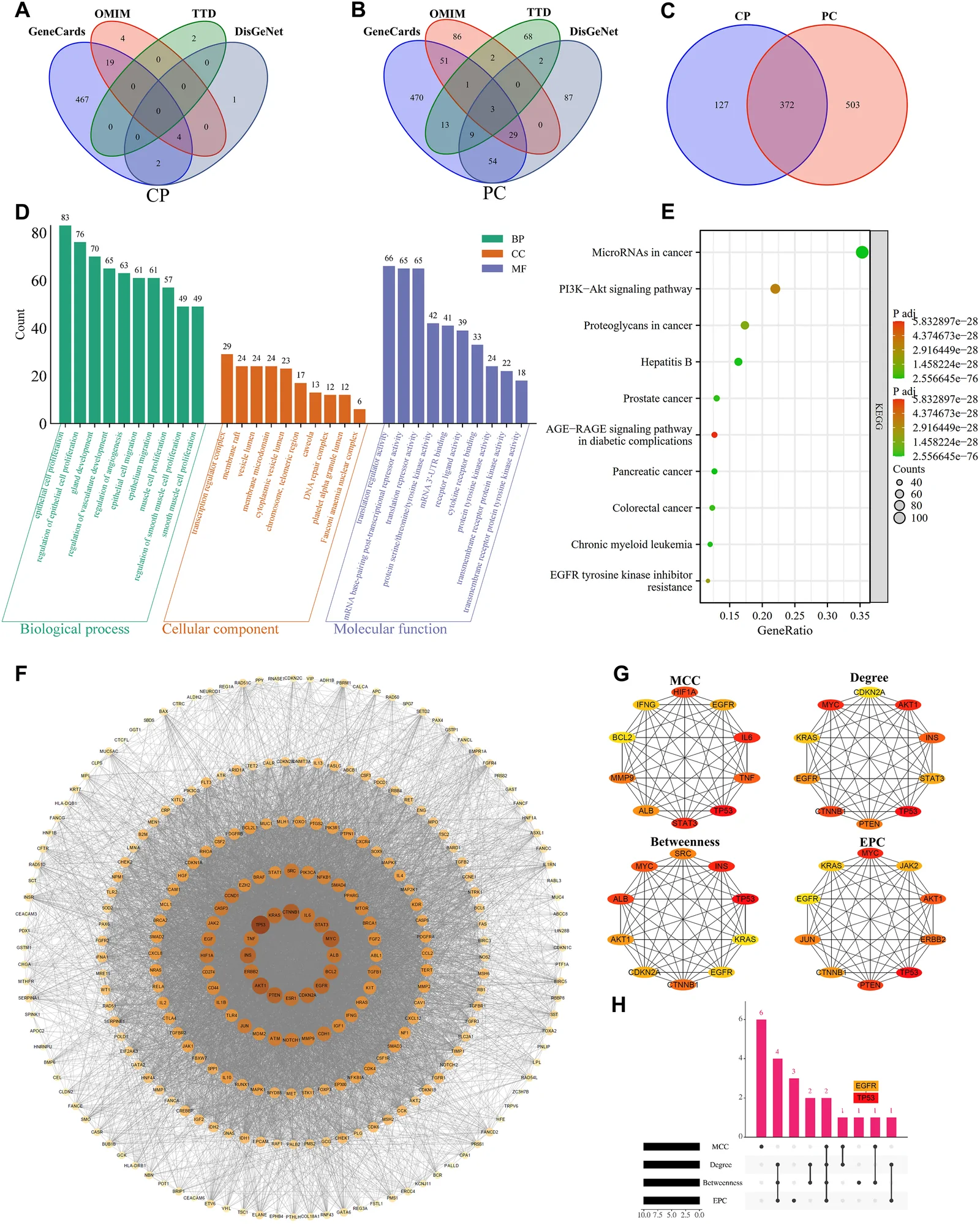
Identification and functional enrichment analysis of CP- and PC-related targets. **(A,B)** Venn diagrams showing CP-related targets **(A)** and PC-related targets **(B)** obtained from the GeneCards, OMIM, TTD, and DisGeNET databases. **(C)** Intersection analysis of CP- and PC-related targets. **(D)** GO functional enrichment analysis of the 372 overlapping targets. **(E)** KEGG pathway enrichment analysis of the 372 overlapping targets. **(F)** PPI network of overlapping genes. **(G)** Screening of hub genes based on the MCC, Degree, Betweenness, and EPC algorithms. **(H)** UpSet plot showing the intersection of hub genes screened by different algorithms.

By integrating the GeneCards, OMIM, TTD, and DisGeNET databases, potential target genes related to CP and PC were identified (**Figures 3A,B**). Venn diagram analysis showed that CP and PC shared 372 overlapping target genes (**Figure 3C**). GO and KEGG enrichment analyses were then performed on these overlapping genes. GO analysis revealed significant enrichment across BP, CC, and MF categories (**Figure 3D**). Specifically, the genes were significantly enriched in epithelial cell proliferation in BP, DNA repair complex in CC, and mRNA base-pairing post-transcriptional repressor activity in MF. KEGG enrichment analysis showed that the overlapping genes were mainly enriched in pancreatic cancer, the PI3K-Akt signaling pathway, colorectal cancer, prostate cancer, and the AGE-RAGE signaling pathway in diabetic complications (**Figure 3E**). A PPI network of the 372 overlapping genes was further constructed using the STRING database (**Figure 3F**). Hub genes were screened in Cytoscape using the MCC, Degree, Betweenness, and EPC algorithms in the CytoHubba plugin. EGFR and TP53 were jointly identified by all four algorithms as key hub genes associated with CP and PC (**Figures 3G,H**).

### 3.2 Network pharmacology analysis of common potential targets of ISO and CP-PC

**Figure 4:**
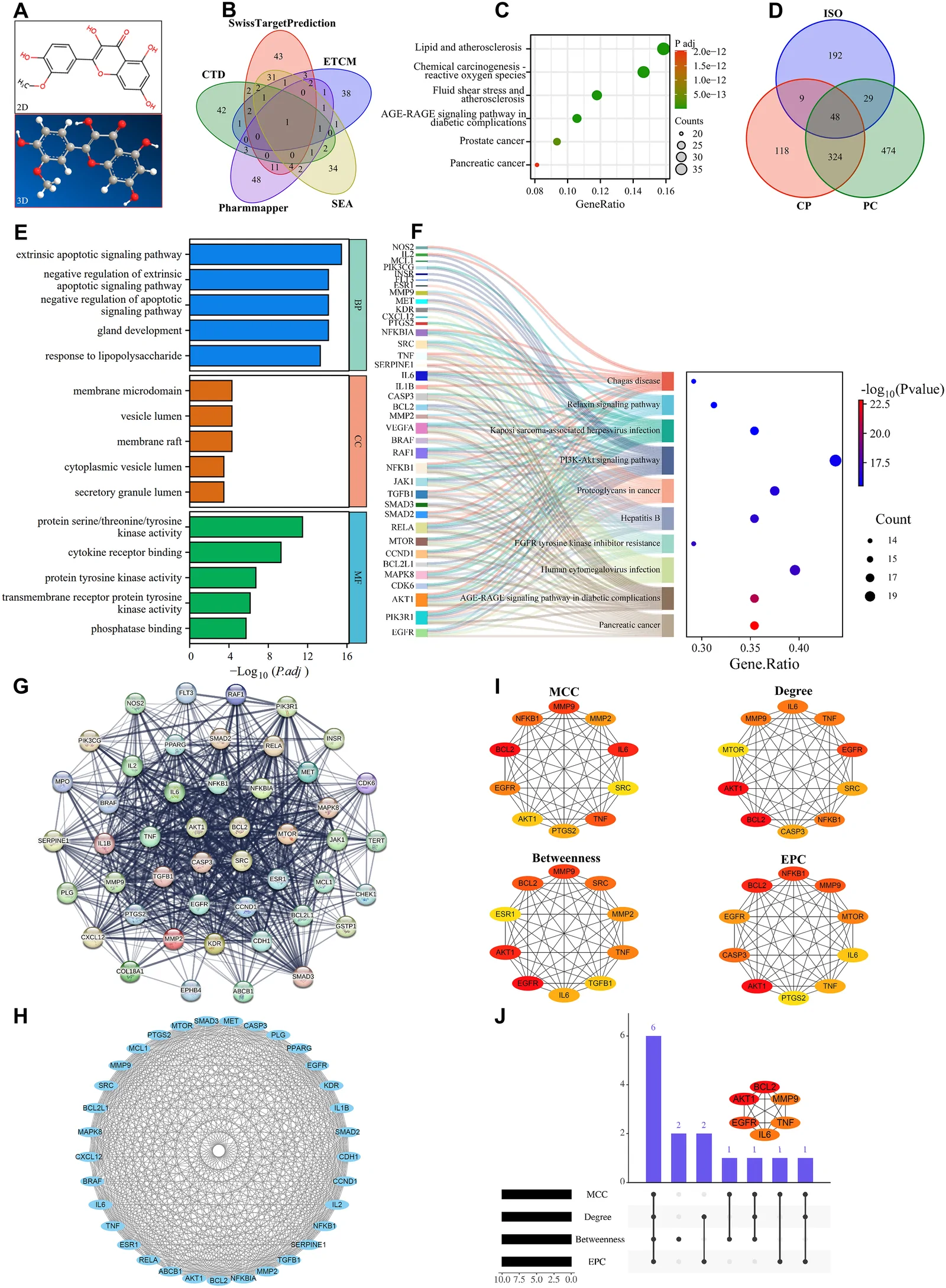
Screening and functional enrichment analysis of potential targets of ISO against CP-PC-related pathological processes. **(A)** Two-dimensional and three-dimensional structures of ISO from the PubChem database. **(B)** Integrated analysis of potential ISO targets from five drug target databases. **(C)** KEGG pathway enrichment analysis of ISO targets. **(D)** Intersection analysis of ISO targets and CP-PC common targets. **(E)** GO functional enrichment analysis of the 48 overlapping targets. **(F)** KEGG enrichment analysis of the 48 overlapping targets. **(G)** PPI network of the 48 overlapping targets. **(H)** Key functional modules in the PPI network identified by the MCODE plugin. **(I)** Top 10 hub genes in the PPI network screened by the CytoHubba plugin. **(J)** UpSet plot showing the hub genes jointly identified by the Degree, EPC, MCC, and Betweenness algorithms.

The two-dimensional and three-dimensional chemical structures of ISO are shown in **Figure 4A**. By integrating the CTD, PharmMapper, ETCM, SEA, and SwissTargetPrediction databases, a total of 278 potential ISO targets were obtained (**Figure 4B**). KEGG enrichment analysis showed that ISO-related targets were involved in the regulation of various diseases and signaling pathways, suggesting broad biological effects (**Figure 4C**). The intersection of ISO targets and CP-PC common targets yielded 48 candidate ISO-related targets (**Figure 4D**). Functional enrichment analysis of these 48 overlapping targets identified 2,564 significantly enriched terms, including 2,398 GO terms and 166 KEGG pathways. GO analysis showed that these targets were significantly enriched in BP, CC, and MF categories, with the top five enriched terms shown in **Figure 4E**. KEGG analysis revealed that pancreatic cancer was the top-ranked enriched pathway, suggesting that ISO may be associated with the regulation of CP- and PC-related molecular networks on CP-PC-related pathological processes by suggesting a potential link with PC-related pathways (**Figure 4F**). Subsequently, the PPI network of the 48 overlapping targets was constructed using the STRING database and imported into Cytoscape for topological analysis (**Figure 4G**). Highly interconnected functional modules were identified using the MCODE plugin, resulting in 33 candidate hub genes (**Figure 4H**). The Degree, EPC, MCC, and Betweenness algorithms in the CytoHubba plugin were further used to screen the top 10 key genes (**Figure 4I**). UpSet plot analysis showed that BCL2, AKT1, IL6, EGFR, MMP9, and TNF were jointly identified by all four algorithms as hub genes involved in the effects of ISO on CP-PC (**Figure 4J**).

### 3.3 Expression, prognostic relevance, and associations with estimated immune-cell infiltration of hub genes in PC

**Figure 5:**
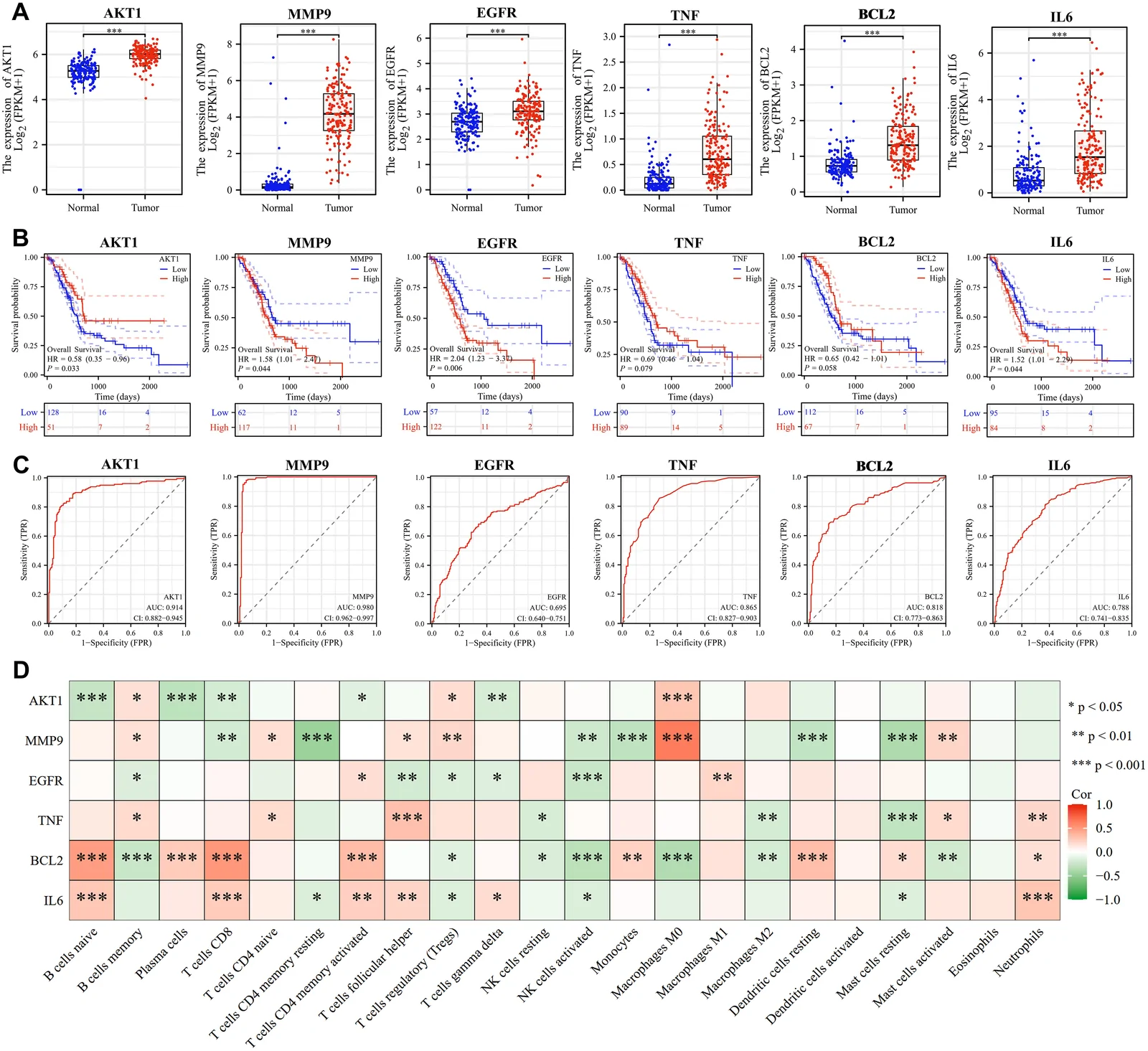
Expression, prognostic relevance, diagnostic performance, and associations with estimated immune-cell infiltration of hub genes in PC. **(A)** mRNA expression levels of AKT1, MMP9, EGFR, TNF, BCL2, and IL6 in PC tissues and normal tissues. **(B)** Kaplan–Meier survival analysis of the association between hub gene expression and OS in patients with PC. **(C)** ROC curve analysis of the hub genes. **(D)** Heatmap showing correlations between hub gene expression and immune cell infiltration. *P < 0.05, **P < 0.01, ***P < 0.001.

To further evaluate the potential biological relevance of the six hub genes in PC, we analyzed their expression, prognostic associations, discriminatory performance, and relationships with computationally estimated immune-cell infiltration. Analysis of the TCGA-PAAD and GTEx datasets showed that the mRNA expression levels of AKT1, MMP9, EGFR, TNF, BCL2, and IL6 were significantly higher in pancreatic cancer tissues than in normal pancreatic tissues (**Figure 5A**). Kaplan–Meier survival analysis showed that higher AKT1 expression was associated with longer OS, whereas higher MMP9, EGFR, and IL6 expression was associated with shorter OS (**Figure 5B**). These findings suggest that the prognostic associations of the candidate genes may differ according to their biological context and should not be interpreted as evidence of a uniform tumor-promoting role. ROC curve analysis indicated that the six hub genes showed varying degrees of ability to distinguish PC tissues from normal pancreatic tissues within the analyzed datasets (**Figure 5C**). However, because the analyses were based on retrospective public datasets and were not validated in an independent external cohort, their potential diagnostic value should be regarded as preliminary. CIBERSORTx-based immune deconvolution further identified correlations between the expression levels of the six hub genes and the estimated fractions of multiple immune-cell populations (**Figure 5D**). Among these associations, activated natural killer cells and several T-cell subsets, including CD4^+^ T cells, CD8^+^ T cells, and T helper 2 cells, were correlated with the expression of multiple hub genes. Importantly, these analyses were based on computational deconvolution of bulk transcriptomic data and did not involve ISO-treated samples or direct measurements of immune-cell abundance or function. Therefore, the results indicate associations between candidate-gene expression and estimated immune-cell composition in PC, but do not demonstrate that ISO regulates immune-cell infiltration, activation, recruitment, polarization, or antitumor function.

### 3.4 Molecular docking analysis of the interactions between ISO and hub targets

**Figure 6:**
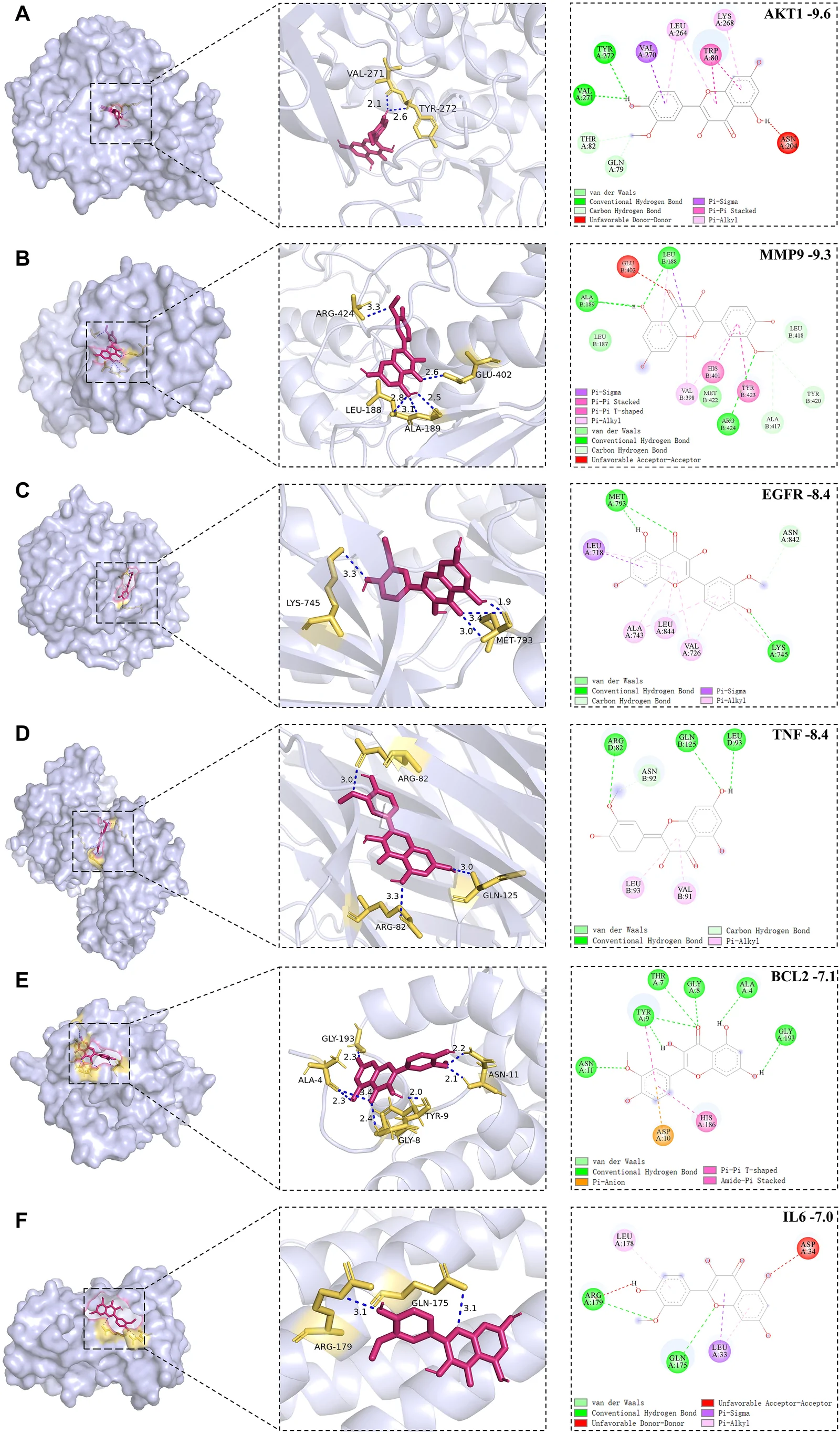
Two-dimensional and three-dimensional structures of the binding sites between ISO and six target proteins. **(A)** AKT1. **(B)** MMP9. **(C)** EGFR. **(D)** TNF. **(E)** BCL2. **(F)** IL6. ISO is shown in red, and target proteins are shown in light blue. Binding energies are labeled in kcal/mol.

To preliminarily evaluate the potential binding interactions between ISO and the hub target proteins, molecular docking was performed to analyze the predicted binding modes of ISO with AKT1, MMP9, EGFR, TNF, BCL2, and IL6. The results showed that ISO exhibited favorable predicted binding affinities with all six hub proteins, with binding energies of −9.6 kcal/mol for AKT1, −9.3 kcal/mol for MMP9, −8.4 kcal/mol for EGFR, −8.4 kcal/mol for TNF, −7.1 kcal/mol for BCL2, and −7.0 kcal/mol for IL6 (**Figures 6A–F**). Among them, ISO showed the lowest predicted binding energy with AKT1, suggesting a relatively favorable docking interaction between ISO and AKT1 in this computational model (**Figure 6A**). Further analysis of intermolecular interactions indicated that ISO may interact with these target proteins through conventional hydrogen bonds, carbon–hydrogen bonds, π-alkyl interactions, and other non-covalent interactions (**Figures 6A–F**). Because redocking validation using co-crystallized ligands was not performed, these docking results should be interpreted as preliminary structural predictions rather than definitive evidence of direct ISO–target binding.

### 3.5 Molecular dynamics simulation analysis of the predicted stability of ISO–target protein complexes

**Figure 7:**
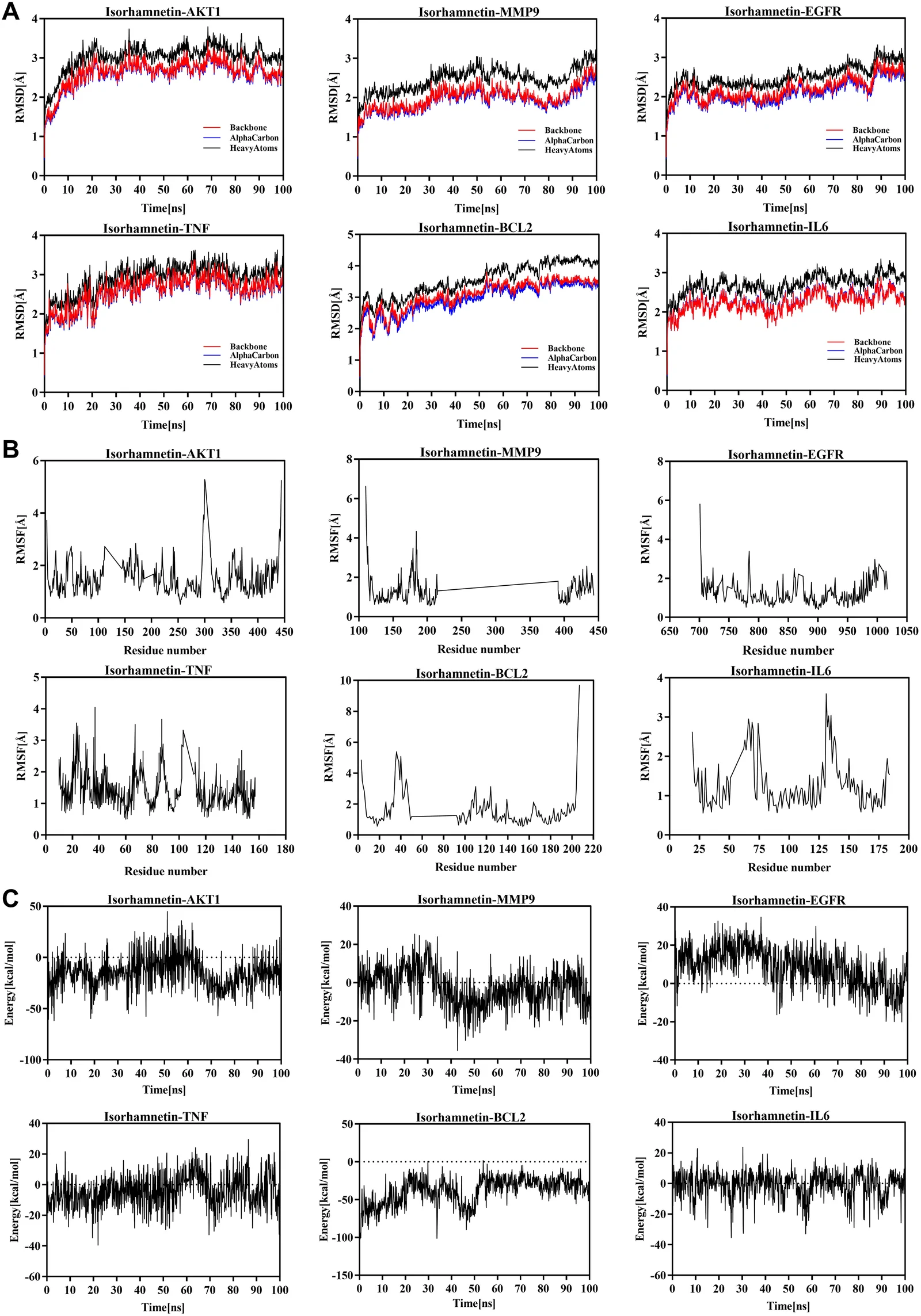
Molecular dynamics simulation analysis of ISO–hub target protein complexes. **(A)** RMSD curves of each ISO–target protein complex during the 100 ns simulation. **(B)** RMSF analysis of residue flexibility in each target protein. **(C)** Energy changes of each ISO–target protein complex during simulation.

To further validate the dynamic stability of ISO–hub target protein complexes, 100 ns all-atom MDS was performed using YASARA software. After simulation, trajectory files were analyzed, and RMSD, RMSF, and energy changes were calculated. RMSD analysis showed that during the 100 ns simulation, the RMSD values of each ISO–target protein complex fluctuated slightly overall and gradually stabilized, indicating favorable conformational stability of the complexes in the simulation system (**Figure 7A**). RMSF analysis further reflected the flexibility changes of different protein residues during binding. Most residues exhibited low fluctuation amplitudes, while only a few local regions showed relatively high flexibility (**Figure 7B**). In addition, energy change analysis showed that the overall energy of each complex remained at a relatively low level and was relatively stable throughout the simulation (**Figure 7C**). Collectively, the RMSD, RMSF, and energy profiles provided preliminary support for the conformational stability of the predicted ISO–target complexes during the 100 ns simulations. However, because radius of gyration, hydrogen bond occupancy, MM-PBSA/MM-GBSA binding free energy, and principal component analyses were not performed, the stability of these complexes requires further computational validation.

### 3.6 MR analysis results

**Figure 8:**
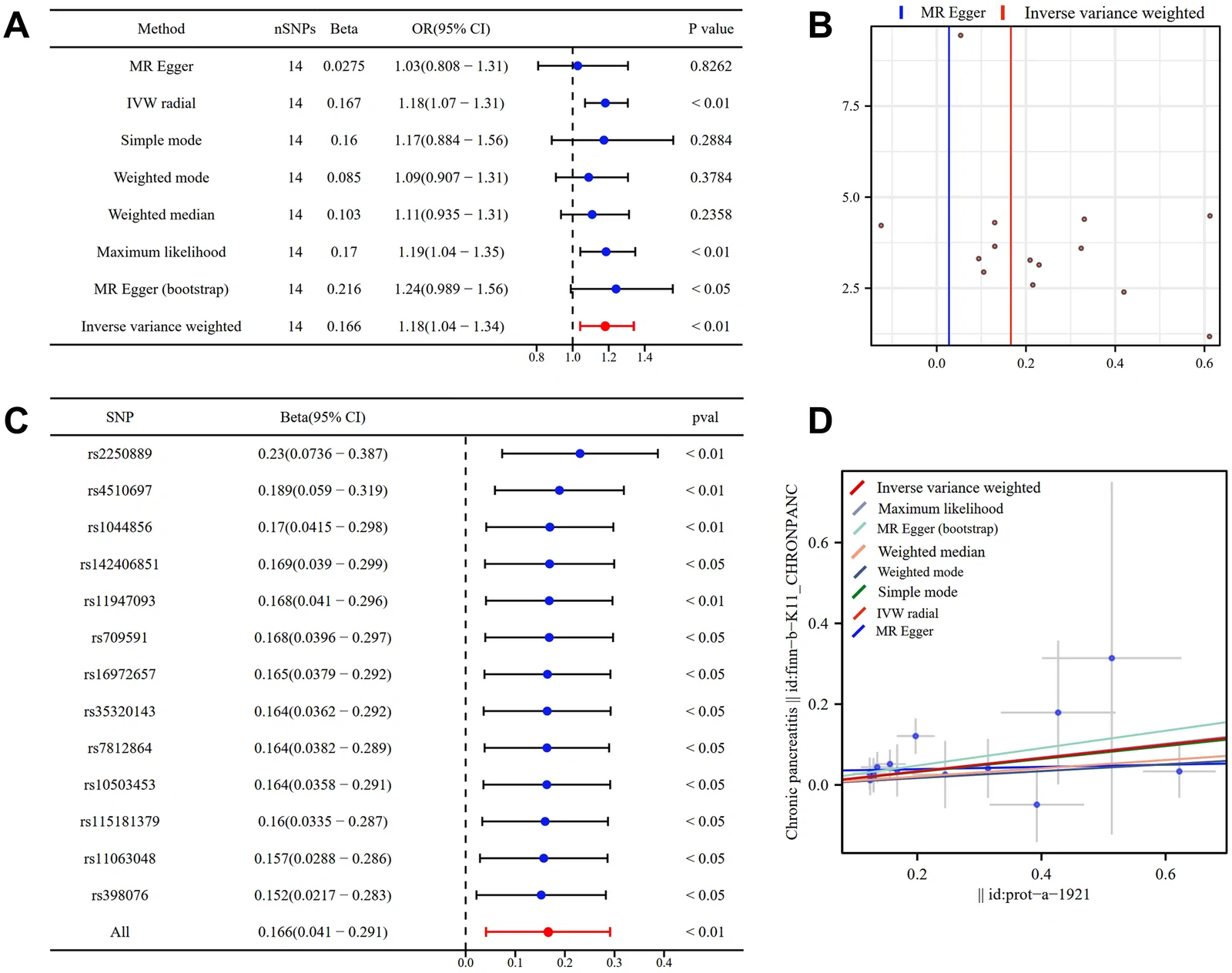
Visualization of MR analysis results. **(A)** Forest plot showing the association between genetically proxied circulating MMP9 protein level and CP risk assessed by different MR methods. **(B)** Funnel plot of the MR analysis. **(C)** Leave-one-out analysis evaluating the influence of individual SNPs on the MR estimate. **(D)** Scatter plot showing MR estimates for the association between MMP9 protein level and CP risk.

MR analysis was performed for all 12 prespecified combinations of the six hub-protein traits (BCL2, AKT1, EGFR, IL6, MMP9, and TNF) with the two outcomes (CP and PC). Complete primary IVW estimates are provided in **Supplementary Table 3**. Among the 12 comparisons, only genetically proxied MMP9 protein level–CP met the nominal *P* < 0.05 criterion (14 SNPs; OR = 1.181, 95% CI: 1.042–1.338, nominal *P* = 0.0093), whereas the other 11 IVW estimates had nominal *P* values > 0.05. Because no formal multiplicity adjustment was applied in this exploratory MR analysis, the MMP9–CP finding is described as a nominal, hypothesis-generating association rather than definitive causal evidence. Similar positive trends for MMP9–CP were observed using the radial IVW and maximum likelihood methods (**Figure 8A**). For the MMP9–CP analysis, 14 SNPs were retained after exposure–outcome harmonization. Their F-statistics ranged from 20.86 to 113.09 and were all > 10 (**Supplementary Table 4**). Cochran’s Q test showed no significant heterogeneity (MR-Egger Q-test *P* = 0.8842; IVW Q-test *P* = 0.8211; **Supplementary Table 5**), and the MR-Egger intercept test did not indicate significant directional horizontal pleiotropy (intercept = 0.0335, SE = 0.0253, *P* = 0.2096; **Supplementary Table 6**). Leave-one-out analysis showed that sequential removal of individual SNPs did not materially change the MMP9–CP estimate (**Figure 8C**), while the funnel and scatter plots supported the overall consistency of the estimates (**Figures 8B,D**). These sensitivity analyses support the internal stability of the MMP9–CP signal, while its exploratory status and multiplicity should be considered when interpreting the result.

### 3.7 Effects of ISO on PC cell viability and related protein expression

To validate the inhibitory effect of ISO on PC cells, CCK-8 assays were performed to detect changes in the viability of PANC-1 and BXPC-3 cells after treatment with different concentrations of ISO, namely 0, 20, 40, and 80 μM. The results showed that cell viability decreased significantly in both PC cell lines with increasing ISO concentrations, exhibiting a clear dose-dependent pattern (*P* < 0.001) (**Figures 1A, B**). After treatment with 80 μM ISO, the survival rates of both PANC-1 and BXPC-3 cells decreased to relatively low levels, indicating that ISO significantly inhibited PC cell proliferation. To further identify key molecular targets associated with the anti-PC effects of ISO, intersection analysis was performed based on the most significantly enriched BP, CC, and MF GO terms and KEGG pathways, together with CP-PC hub genes and ISO-CP-PC-related hub genes. The results showed that EGFR was the only overlapping gene, suggesting that EGFR may be an important target through which ISO intervenes in CP-PC-related pathological processes (**Figure 1C**). Western blot analysis was then performed to detect the protein expression levels of EGFR, AKT1, BCL2, and TNFα. The results showed that as the concentration of ISO increased, the protein expression levels of EGFR, AKT1, BCL2, and TNFα were markedly downregulated in PANC-1 and BXPC-3 cells (**Figures 1D–G**). Densitometric analysis was consistent with the changes in protein bands, indicating that ISO treatment was associated with reduced total EGFR, AKT1, BCL2, and TNFα protein abundance in PC cells. Because phosphorylated EGFR and phosphorylated AKT were not detected in this study, these results cannot directly demonstrate inhibition of EGFR/AKT pathway activation.

### 3.8 Effects of ISO on PC cell wound closure and apoptosis

To further investigate the effects of ISO on the malignant biological behaviors of PC cells, wound-healing assays were performed to assess changes in wound closure in PANC-1 and BXPC-3 cells. The results showed that compared with the control group, wound closure ability was markedly reduced in both cell lines after ISO treatment. As ISO concentration increased, the wound closure rate gradually decreased (P < 0.001) (**Figures 2A, B**). However, because ISO also reduced cell viability in the CCK-8 assay, the decreased wound closure may reflect combined effects on cell proliferation and migration rather than proliferation-independent migration inhibition alone. Therefore, these data should be interpreted cautiously, and further validation using proliferation-controlled migration assays, such as Transwell migration assays, is required. Annexin V-FITC/PI double-staining flow cytometry was then used to detect the effect of ISO on PC cell apoptosis. The results showed that ISO treatment significantly increased the apoptosis rates of both PANC-1 and BXPC-3 cells in a concentration-dependent manner (P < 0.001) (**Figures 2C, D**). Among the treatment groups, the 80 μM ISO group showed the highest apoptosis rate.

## 4 Discussion

CP is a continuously progressive inflammatory disease of the pancreas, whereas PC is one of the most aggressive malignancies of the digestive system and is associated with extremely poor prognosis (2, 29, 30). CP not only substantially impairs patients’ quality of life but also represents a key risk factor for PC development. Current clinical treatment strategies for CP and PC, including surgery and chemotherapy, are limited by difficulties in early diagnosis, unsatisfactory therapeutic efficacy, and the frequent development of drug resistance (2, 31, 32). Therefore, elucidating the pathological mechanisms underlying the transition from CP to PC and identifying novel intervention targets are urgently needed to improve patient prognosis.

In this study, network pharmacology, molecular docking, molecular dynamics simulation, MR analysis, and *in vitro* cellular experiments were integrated to systematically investigate the potential molecular mechanisms by which the natural flavonoid ISO intervenes in CP-PC-related pathological processes. First, bioinformatics analysis was used to identify common targets of CP and PC and potential targets of ISO, leading to the identification of a series of hub genes, including BCL2, AKT1, IL6, EGFR, MMP9, and TNF (33). Subsequent computational simulation and genetic analyses provided preliminary support for ISO–target binding and identified a hypothesis-generating association between genetically proxied MMP9 protein level and CP risk. Cellular experiments further confirmed that ISO reduced PC cell viability and wound closure and induced apoptosis, with these effects being associated with the downregulation of key proteins such as EGFR and AKT1. Previous studies have reported that ISO can suppress pancreatic cancer cell proliferation and induce apoptosis, supporting its potential antitumor activity. Our findings are consistent with these observations, as ISO reduced PC cell viability, decreased wound closure, and promoted apoptosis in PANC-1 and BXPC-3 cells. However, the present study extends prior work by integrating CP- and PC-associated targets, immune-infiltration signatures, structural simulations, and MR-based causal inference. This broader framework suggests that ISO may be linked not only to tumor-cell-autonomous processes but also to inflammation-associated molecular networks. Nevertheless, unlike direct pathway-intervention studies, our evidence remains partly predictive, and the proposed mechanisms require validation using pathway-specific, immune-functional, and inflammatory pancreatic models.

Our bioinformatics analysis integrated multiple databases and systematically revealed the shared pathological basis of CP and PC at the molecular level. The 372 overlapping disease-associated genes identified in this study were significantly enriched in key biological processes such as epithelial cell proliferation and DNA repair, as well as in pathways including the PI3K-Akt signaling pathway. Notably, EGFR and TP53 were consistently identified as hub genes connecting CP and PC by multiple topological algorithms. This finding has strong biological plausibility. EGFR signaling plays a central role in driving cell proliferation and survival, and its aberrant activation is a common feature of various solid tumors, including PC (34–36). TP53, as a key tumor suppressor gene, contributes to increased genomic instability when functionally inactivated and is closely associated with the transition from chronic inflammation to malignant transformation (37–39). The PI3K-Akt pathway, identified as a key pathway in our enrichment analysis, may represent an important molecular bridge linking the chronic inflammatory microenvironment of CP with uncontrolled proliferation in PC, thereby providing new insights into the transformation mechanism between the two diseases.

Further network pharmacology analysis predicted the potential targets through which ISO may intervene in CP-PC pathological processes. ISO has been widely reported to possess multiple pharmacological activities, including anti-inflammatory, antioxidant, and antitumor effects (14). Our analysis identified 48 potential ISO targets overlapping with overlapping CP- and PC-associated genes, among which BCL2, AKT1, IL6, EGFR, MMP9, and TNF were identified as six hub genes. The prioritization of these six genes was based on both topological robustness and biological relevance. These genes were repeatedly identified by multiple network algorithms rather than by a single centrality measure, reducing the likelihood that their selection was driven by one network property alone. Biologically, EGFR and AKT1 are involved in growth factor-related survival signaling (40, 41), BCL2 is a central apoptosis-regulatory molecule (42), TNF and IL6 are key inflammatory cytokines (43), and MMP9 participates in extracellular matrix remodeling and inflammatory cell infiltration (44). Thus, these six targets collectively cover several major processes involved in CP-associated pancreatic tumor biology, including inflammation, proliferation, survival, apoptosis resistance, and tissue remodeling. Interestingly, the core pathway enriched among predicted ISO targets was the pancreatic cancer pathway, which is highly related to the PI3K-Akt pathway enriched among overlapping CP- and PC-associated genes. Together, these findings suggest a potential multitarget regulatory network through which ISO may be linked to inflammation-associated pancreatic tumor biology.

To evaluate the clinical significance of these hub targets in PC, public databases were used for in-depth analysis. The results showed that AKT1, MMP9, EGFR, TNF, BCL2, and IL6 were all significantly upregulated at the mRNA level in PC tissues, and high expression of several genes, including MMP9, EGFR, and IL6, was associated with poor patient prognosis, suggesting their active involvement in disease progression (45–47). One noteworthy finding was that high AKT1 mRNA expression was associated with better OS, which differed from the adverse prognostic trends observed for MMP9, EGFR, and IL6. This apparently contradictory result should be interpreted cautiously. AKT signaling activity is not determined solely by AKT1 mRNA abundance or total AKT protein level, but is largely dependent on phosphorylation status, especially activation-associated phosphorylation of AKT. Therefore, high AKT1 transcript expression in public survival datasets does not necessarily indicate enhanced AKT pathway activation. Moreover, pancreatic cancer is molecularly heterogeneous, and different molecular subtypes may display distinct biological behaviors, stromal compositions, immune contexts, and pathway dependencies. AKT1 expression may therefore reflect subtype-specific transcriptional states or tumor microenvironmental features rather than a uniformly oncogenic phenotype. Because we were unable to perform a robust TCGA-based stratified survival analysis according to molecular subtype or phospho-AKT status in the present study, this issue remains unresolved and should be addressed in future studies integrating transcriptomic, proteomic, phosphoproteomic, and molecular subtype information. In addition, immune infiltration analysis revealed that the expression of these hub genes was significantly correlated with the infiltration levels of activated natural killer cells, CD8^+^ T cells, and other immune cells. These findings suggest a potential association between ISO-related hub targets and immune infiltration patterns in PC. However, this analysis was based on transcriptome-derived immune deconvolution and cannot determine whether ISO directly modulates immune cell recruitment, activation, or antitumor immune function. Therefore, the immunomodulatory role of ISO in the pancreatic tumor microenvironment remains hypothetical and requires validation using immune-cell co-culture systems, flow cytometry, cytokine profiling, animal models, or clinical specimens.

At the cellular level, our *in vitro* experiments directly validated the anti-PC activity of ISO and partially clarified its mechanism of action. ISO decreased wound closure in PANC-1 and BXPC-3 cells, although this result may reflect combined effects on proliferation and migration. Western blot results further confirmed that ISO treatment downregulated the protein expression of EGFR, AKT1, BCL2, and TNFα. These *in vitro* data partially support the bioinformatics predictions by showing that ISO reduced total EGFR, AKT1, BCL2, and TNFα protein levels in PC cells. However, because only total EGFR and AKT1 proteins were measured, and phosphorylated EGFR and phosphorylated AKT were not examined, the present results cannot determine whether ISO directly inhibits EGFR/AKT pathway activation. Therefore, the relationship between ISO treatment and EGFR/AKT signaling activity should be regarded as suggestive rather than definitive.

To move beyond correlative associations, MR analysis was used to explore potential causal relationships between genetically proxied hub-protein levels and disease. In the complete 12-combination IVW screen, MMP9–CP was the only comparison with a nominal *P* < 0.05 (OR = 1.181, 95% CI: 1.042–1.338, nominal *P* = 0.0093), indicating that genetically proxied higher circulating MMP9 protein levels were associated with increased CP risk. Because this analysis was exploratory and no formal multiplicity adjustment was applied, the MMP9–CP result should be regarded as a hypothesis-generating genetic signal rather than definitive causal evidence. MMP9 may aggravate chronic injury and fibrosis by degrading the basement membrane and extracellular matrix surrounding pancreatic acini, disrupting tissue structural integrity, and promoting inflammatory cell infiltration (48). This finding supports further investigation of MMP9 as a CP-relevant target; however, whether ISO directly regulates MMP9 remains unconfirmed in the present study. The present *in vitro* experiments did not directly examine MMP9 or IL6 protein levels after ISO treatment. This is an important limitation because MMP9 and IL6 were identified as hub targets through network pharmacology and bioinformatics analyses, while MMP9 was further prioritized by the exploratory MR signal. The effects of ISO on MMP9 and IL6 protein abundance, secretion, or activity were not directly examined in the present study. Therefore, whether ISO directly regulates MMP9- or IL6-related inflammatory responses remains unconfirmed. Future studies should include WB, ELISA, gelatin zymography for MMP9 activity, and inflammatory cell or pancreatitis models to clarify the effects of ISO on these inflammatory mediators.

Several limitations should be acknowledged. Target identification was dependent on database coverage and cutoff selection, and alternative-threshold sensitivity analyses were not performed. The immune infiltration results were based on computational deconvolution and therefore do not prove direct immune modulation by ISO. Docking and molecular dynamics findings also require further validation, including redocking, binding free-energy estimation, hydrogen bond occupancy, radius of gyration, and principal component analyses. The exploratory MR screen identified a nominal MMP9–CP association, but no formal multiplicity adjustment was applied; the inference also remains dependent on instrumental-variable strength and pleiotropy/heterogeneity assumptions. Experimentally, validation was limited to pancreatic cancer cell lines and lacked inflammatory pancreatic models, immune-cell assays, MMP9/IL6 protein detection, EGFR/AKT phosphorylation analysis, downstream PI3K/AKT markers, and caspase-related markers. In addition, Transwell migration assays and independent ROC validation were not performed, so the wound-healing and diagnostic findings should be regarded as preliminary.

## 5 Conclusion

In conclusion, this study provides an integrated, hypothesis-generating framework suggesting that ISO may be associated with CP- and PC-related molecular networks, inflammation-related and immune-infiltration-associated signatures, apoptosis, epithelial proliferation, extracellular matrix remodeling, and PI3K-Akt-related pathways. EGFR, AKT1, MMP9, BCL2, TNF, and IL6 were identified as potential molecular nodes linking pancreatic inflammation and tumor-associated biological processes. Cellular experiments showed that ISO reduced PC cell viability, decreased wound closure, promoted apoptosis, and reduced total EGFR, AKT1, BCL2, and TNFα protein abundance. However, the present study did not directly validate immune functional effects, MMP9 or IL6 protein changes, EGFR/AKT phosphorylation status, downstream pathway activation, or proliferation-independent migration. In addition, docking and molecular dynamics findings require further computational and biophysical validation, and ROC-based diagnostic implications require independent external validation. Therefore, these findings should be interpreted cautiously and require further confirmation in inflammatory pancreatic models, immune-cell systems, animal studies, and clinical samples.

## Data Availability

The original contributions presented in the study are included in the article/**Supplementary Material**. Further inquiries can be directed to the corresponding author.
