# Supplementary material for: Integrated analysis of isorhamnetin-associated targets in chronic pancreatitis and pancreatic cancer: immune-infiltration associations and antitumor effects in pancreatic cancer cells
Source: Front Immunol. 2026 Sep 4;17:1902080. doi: 10.3389/fimmu.2026.1902080 (PMC13586020; doi:10.3389/fimmu.2026.1902080)
Supplement: Supplementary file 1 [file Table1.docx]

Supplementary Material

# 1 Supplementary Tables

**Supplementary Table S1.** Databases, platforms, and software used in this study.

| **Category** | **Database / platform / software** | **Version / release information** | **Uniform resource locator** | **Access date** |
| --- | --- | --- | --- | --- |
| Disease target database | GeneCards | Online database | https://www.genecards.org/ | 2025-04-08 |
| Disease target database | DisGeNET | Online database | https://disgenet.com/ | 2025-04-08 |
| Disease target database | Online Mendelian Inheritance in Man (OMIM) | Online database | https://www.omim.org/ | 2025-04-08 |
| Disease target database | Therapeutic Target Database (TTD) | Online database | https://ttd.idrblab.cn/ | 2025-04-08 |
| Drug-target prediction database | Comparative Toxicogenomics Database (CTD) | Online database | <https://ctdbase.org/> | 2025-04-09 |
| Drug-target prediction platform | PharmMapper | Online platform | https://www.lilab-ecust.cn/pharmmapper/ | 2025-04-09 |
| Drug-target prediction database | Encyclopedia of Traditional Chinese Medicine (ETCM) | Online database | http://www.tcmip.cn/ETCM/ | 2025-04-09 |
| Drug-target prediction platform | Similarity Ensemble Approach (SEA) | Online platform | https://sea.bkslab.org/ | 2025-04-09 |
| Drug-target prediction platform | SwissTargetPrediction | Online platform | <https://swisstargetprediction.ch/> | 2025-04-09 |
| PPI database | STRING | Version 12.0 | <https://string-db.org/> | 2025-04-11 |
| Bioinformatics software | Cytoscape | Version 3.9.1 | <https://cytoscape.org/> | 2025-04-11 |
| Cytoscape plugin | CytoHubba | Version 0.1 | <https://apps.cytoscape.org/apps/cytohubba> | 2025-04-11 |
| Cytoscape plugin | MCODE | Version 2.0.3 | <https://apps.cytoscape.org/apps/mcode> | 2025-04-11 |
| R software | R | Version 4.3.2 | https://www.r-project.org/ | 2025-04-20 |
| R package | clusterProfiler | Version 4.10.0 | <https://bioconductor.org/> | 2025-04-20 |
| R package | ggplot2 | Version 3.4.4 | [https://github.com/tidyverse/ggplot2/](https://github.com/tidyverse/ggplot2) | 2025-04-20 |
| R package | pROC | Version 1.18.0 | <https://cran.r-project.org/package=pROC> | 2025-04-20 |
| R package | TwoSampleMR | Version 0.6.0 | <https://mrcieu.github.io/> | 2025-04-25 |
| Expression database | TCGA-PAAD via GDC Data Portal | Online database | https://portal.gdc.cancer.gov/ | 2025-04-18 |
| Expression database | GTEx | Online database | <https://gtexportal.org/home/> | 2025-04-18 |
| Survival analysis platform | Kaplan–Meier Plotter | Online platform | <https://kmplot.com/analysis/> | 2025-04-20 |
| Immune infiltration algorithm | CIBERSORTx | Online algorithm | <https://cibersortx.stanford.edu/> | 2025-04-22 |
| Chemical structure database | PubChem | Online database | <https://pubchem.ncbi.nlm.nih.gov/> | 2025-06-28 |
| Protein structure database | Protein Data Bank (PDB) | Online database | <https://www.rcsb.org/> | 2025-06-28 |
| Molecular conversion software | Open Babel | Version 2.3.2 | <https://openbabel.org/> | 2025-06-28 |
| Molecular visualization software | PyMOL | Version 2.3.4 | <https://www.pymol.org/> | 2025-06-28 |
| Docking preparation software | AutoDockTools | Version 1.5.7 | <https://autodock.scripps.edu/> | 2025-06-28 |
| Molecular docking software | AutoDock Vina | Version 1.1.2 | <https://vina.scripps.edu/> | 2025-06-28 |
| Molecular visualization software | BIOVIA Discovery Studio | Version 2019 | <https://www.3ds.com/products/biovia/discovery-studio/> | 2025-07-02 |
| Molecular dynamics software | YASARA | Version 10.3.16 | https://www.yasara.org/ | 2025-07-20 |
| GWAS database | IEU OpenGWAS | Online database | https://opengwas.io/ | 2025-08-25 |
| Statistical software | GraphPad Prism | Version 8.0.2 | <https://www.graphpad.com/scientific-software/prism/> | 2026-04-23 |
| Image analysis software | ImageJ | Version 1.54p | <https://imagej.net/ij/> | 2026-04-21 |

**Supplementary Table S2.** Characteristics of the MR exposure and outcome datasets.

| **Trait** | **OpenGWAS ID** | **Source / year** | **Population** | **Sample size / case-control** |
| --- | --- | --- | --- | --- |
| BCL2 protein | prot-a-236 | INTERVAL proteomic GWAS / 2018 | European; males and females | n = 3,301; 10,534,735 SNPs |
| AKT1 protein | prot-a-70 | INTERVAL proteomic GWAS / 2018 | European; males and females | n = 3,301; 10,534,735 SNPs |
| EGFR protein | prot-a-909 | INTERVAL proteomic GWAS / 2018 | European; males and females | n = 3,301; 10,534,735 SNPs |
| IL6 protein | prot-a-1539 | INTERVAL proteomic GWAS / 2018 | European; males and females | n = 3,301; 10,534,735 SNPs |
| MMP9 protein | prot-a-1921 | INTERVAL proteomic GWAS / 2018 | European; males and females | n = 3,301; 10,534,735 SNPs |
| TNF protein | prot-a-3029 | INTERVAL proteomic GWAS / 2018 | European; males and females | n = 3,301; 10,534,735 SNPs |
| Chronic pancreatitis (CP) | finn-b-K11_CHRONPANC | FinnGen / 2021 | European | n = 196,881; 1,737 cases / 195,144 controls |
| Pancreatic cancer (PC) | ebi-a-GCST90018893 | Sakaue et al. / 2021 | European | n = 476,245; 1,196 cases / 475,049 controls |

**Note:** The six exposure datasets are protein-level GWAS summary statistics from the INTERVAL proteomic study (Sun et al., 2018; PMID: 29875488), comprising 3,301 participants of European ancestry and 10,534,735 SNPs per protein dataset. Therefore, the MR exposures represent genetically proxied circulating protein levels rather than mRNA expression. CP, chronic pancreatitis; PC, pancreatic cancer. The CP outcome was obtained from FinnGen, and the PC outcome was obtained from the Sakaue et al. 2021 GWAS (PMID: 34594039).

**Supplementary Table S3.** Complete primary IVW results for all tested hub-protein–outcome combinations.

| **Exposure** | **Outcome** | **SNPs** | **IVW OR (95% CI)** | **Nominal *P* value** |
| --- | --- | --- | --- | --- |
| BCL2 | CP | 8 | 1.055 (0.848–1.313) | 0.6328 |
| BCL2 | PC | 9 | 1.073 (0.845–1.363) | 0.5619 |
| AKT1 | CP | 11 | 0.908 (0.782–1.054) | 0.2055 |
| AKT1 | PC | 13 | 0.947 (0.825–1.087) | 0.4385 |
| EGFR | CP | 8 | 0.924 (0.733–1.165) | 0.5048 |
| EGFR | PC | 9 | 1.041 (0.847–1.279) | 0.7041 |
| IL6 | CP | 12 | 1.045 (0.898–1.216) | 0.5703 |
| IL6 | PC | 12 | 1.006 (0.825–1.227) | 0.9542 |
| MMP9 | CP | 14 | 1.181 (1.042–1.338) | 0.0093 |
| MMP9 | PC | 15 | 1.038 (0.911–1.184) | 0.5738 |
| TNF | CP | 11 | 1.006 (0.847–1.194) | 0.9460 |
| TNF | PC | 13 | 1.151 (0.956–1.387) | 0.1371 |

**Note:** IVW, inverse variance weighted; CP, chronic pancreatitis; PC, pancreatic cancer. All 12 prespecified primary IVW tests are shown. Because this MR component was exploratory and hypothesis-generating, no formal multiple-testing correction was applied; nominal two-sided P values are reported. MMP9–CP was the only comparison with nominal *P* < 0.05 and is therefore described as a hypothesis-generating nominal association rather than definitive causal evidence.

**Supplementary Table S4.** Instrumental variables used for the MR analysis of genetically proxied MMP9 protein level and chronic pancreatitis risk.

| **SNP** | ***P* value exposure** | **SE for exposure** | **chr.exposure** | **pos.exposure** | **beta.exposure** | **id.exposure** | **F-statistic** |
| --- | --- | --- | --- | --- | --- | --- | --- |
| rs11947093 | 3.89E-06 | 0.0531 | 4 | 131087342 | 0.2449 | prot-a-1921 | 21.27 |
| rs115181379 | 3.89E-06 | 0.0924 | 5 | 72479928 | -0.4269 | prot-a-1921 | 21.35 |
| rs4510697 | 2.45E-07 | 0.0761 | 6 | 142709812 | -0.3926 | prot-a-1921 | 26.62 |
| rs10503453 | 4.37E-06 | 0.0269 | 8 | 13402197 | 0.1234 | prot-a-1921 | 21.04 |
| rs7812864 | 4.68E-06 | 0.1122 | 8 | 89843567 | 0.5133 | prot-a-1921 | 20.93 |
| rs142406851 | 3.47E-06 | 0.0676 | 10 | 116853469 | -0.314 | prot-a-1921 | 21.58 |
| rs11063048 | 2.88E-07 | 0.0264 | 12 | 4341158 | -0.1356 | prot-a-1921 | 26.38 |
| rs1044856 | 3.31E-06 | 0.0267 | 13 | 43462422 | 0.1242 | prot-a-1921 | 21.64 |
| rs398076 | 1.02E-09 | 0.0255 | 14 | 88414211 | 0.156 | prot-a-1921 | 37.43 |
| rs16972657 | 4.90E-06 | 0.0367 | 15 | 81318832 | -0.1676 | prot-a-1921 | 20.86 |
| rs35320143 | 3.72E-06 | 0.028 | 16 | 66638245 | -0.1295 | prot-a-1921 | 21.39 |
| rs709591 | 5.01E-07 | 0.0258 | 17 | 38175561 | 0.1299 | prot-a-1921 | 25.35 |
| rs62143194 | 1.05E-10 | 0.0306 | 19 | 54319624 | 0.1974 | prot-a-1921 | 41.62 |
| rs2250889 | 2.09E-26 | 0.0585 | 20 | 44642406 | -0.6221 | prot-a-1921 | 113.09 |

**Note:** SNPs associated with MMP9 protein level were selected as instrumental variables. F-statistics were calculated as β²/SE² based on the SNP–exposure association. SNPs with F-statistics > 10 were considered sufficiently strong instruments.

**Supplementary Table S5.** Heterogeneity assessment of the MR analysis using Cochran’s Q test.

| **Exposure** | **Outcome** | **Method** | **Q** | **Q-df** | **Q-pvalue** |
| --- | --- | --- | --- | --- | --- |
| id:prot-a-1921 | Chronic pancreatitis (id:finn-b-K11_CHRONPANC) | MR Egger | 6.5773 | 12 | 0.8842 |
| id:prot-a-1921 | Chronic pancreatitis (id:finn-b-K11_CHRONPANC) | IVW radial | 8.2131 | 13 | 0.8294 |
| id:prot-a-1921 | Chronic pancreatitis (id:finn-b-K11_CHRONPANC) | Maximum likelihood | 8.2102 | 13 | 0.8296 |
| id:prot-a-1921 | Chronic pancreatitis (id:finn-b-K11_CHRONPANC) | Unweighted regression | 0.0847 | 13 | 1.0000 |
| id:prot-a-1921 | Chronic pancreatitis (id:finn-b-K11_CHRONPANC) | Inverse variance weighted | 8.3352 | 13 | 0.8211 |

**Note:** Heterogeneity among instrumental variables was assessed using Cochran’s Q test. A Q-test P value < 0.05 was considered indicative of significant heterogeneity.

**Supplementary Table S6.** Horizontal pleiotropy assessment of the MR analysis using the MR-Egger intercept test.

| **Exposure** | **Outcome** | **Method** | **Intercept** | **SE** | **P value** |
| --- | --- | --- | --- | --- | --- |
| \|\| id:prot-a-1921 | Chronic pancreatitis \|\| id:finn-b-K11_CHRONPANC | MR Egger | 0.0335 | 0.0253 | 0.2096 |

**Note:** Directional horizontal pleiotropy was assessed using the MR-Egger intercept test. A P value < 0.05 was considered indicative of significant directional horizontal pleiotropy.
